# Artificial intelligence predicts recurrent autoimmune hepatitis after liver transplantation in a multicenter cohort study

**DOI:** 10.1097/HC9.0000000000001004

**Published:** 2026-07-27

**Authors:** Mamatha Bhat, Yingji Sun, Praveen Manickavel, Saba Maleki, Vincenzo Ronca, Bettina E. Hansen, Gideon Hirschfield, Saleh Elwir, Mohamad Alsaed, Piotr Milkiewicz, Maciej K. Janik, Hanns-Ulrich Marschall, Maria Antonella Burza, Cumali Efe, Ali Rıza Calışkan, Murat Harputluoglu, Gökhan Kabaçam, Débora Terrabuio, Fernanda de Quadros Onofrio, Nazia Selzner, Alan Bonder, Albert Parés, Laura Llovet, Murat Akyıldız, Cigdem Arikan, Michael P. Manns, Richard Taubert, Anna-Lena Weber, Thomas D. Schiano, Brandy Haydel, Piotr Czubkowski, Piotr Socha, Natalia Ołdak, Nobuhisa Akamatsu, Atsushi Tanaka, Cynthia Levy, Eric F. Martin, Aparna Goel, Mai Sedki, Irena Jankowska, Toru Ikegami, Maria Rodriguez, Martina Sterneck, Christina Weiler-Normann, Christoph Schramm, Maria Francesca Donato, Ansgar Lohse, Raul J. Andrade, Vilas R. Patwardhan, Bart van Hoek, Maaike Biewenga, Andreas E. Kremer, Yoshihide Ueda, Mark Deneau, Mark Pedersen, Marlyn J. Mayo, Annarosa Floreani, Patrizia Burra, Maria Francesca Secchi, Benedetta Terziroli Beretta-Piccoli, Marco Sciveres, Giuseppe Maggiore, Syed-Mohammed Jafri, Dominique Debray, Muriel Girard, Florence Lacaille, Ynto S. de Boer, Ana Lleo, Andrew L. Mason, Michael Heneghan, Ye Htun Oo, Ellina Lytvyak, Aldo J. Montano-Loza

**Affiliations:** 1 Ajmera Transplant Center, University Health Network, Toronto, Ontario, Canada; 2 Division of Gastroenterology, Faculty of Medicine, University of Toronto, Toronto, Ontario, Canada; 3 Toronto General Hospital Research Institute, University Health Network, Toronto, Ontario, Canada; 4 Center for Liver Research and NIHR Birmingham Biomedical Research Centre (BRC), Institute of Immunology and Immunotherapy, University of Birmingham; University Hospitals Birmingham NHS Foundation Trust, Birmingham, UK; 5 Toronto Center for Liver Disease, University Health Network, University of Toronto, Toronto, Ontario, Canada; 6 Erasmus MC University Medical Center, Rotterdam, The Netherlands; 7 Baylor University Medical Center, Dallas, Texas, USA; 8 Liver and Internal Medicine Unit, Medical University of Warsaw, Warsaw, Poland; 9 Sahlgrenska University Hospital, Gothenburg, Sweden; 10 Department of Gastroenterology, Harran University Hospital, Şanlıurfa, Turkey; 11 Department of Gastroenterology, Adiyaman University School of Medicine, Adiyaman, Turkey; 12 Department of Gastroenterology, Inönü University School of Medicine, Malatya, Turkey; 13 Clinic of Gastroenterology and Liver Transplantation, Guven Hospital Ankara, Turkey; 14 Department of Gastroenterology, University of São Paulo School of Medicine, São Paulo, Brazil; 15 Beth Israel Deaconess Medical Center, Harvard Medical School, Boston, Massachusetts, USA; 16 Liver Unit, Hospital Clínic, University of Barcelona, IDIBAPS, CIBERehd, Barcelona, Spain; 17 Koç University School of Medicine, Department of Gastroenterology and Liver Transplantation Center, Istanbul, Turkey; 18 Division of Pediatric Gastroenterology and Hepatology, Organ Transplantation Center, Koc University School of Medicine, Koc University Research Center for Translational Medicine (KUTTAM), Istanbul, Turkey; 19 European Reference Network on Hepatological Diseases (ERN RARE-LIVER), Department of Gastroenterology, Hepatology and Endocrinology, Hannover Medical School, Hannover, Germany; 20 Recanati/Miller Transplantation Institute/Division of Liver Diseases, Mount Sinai Medical Center, New York, New York, USA; 21 Department of Gastroenterology, Hepatology, Nutritional Disorders and Pediatrics, The Children’s Memorial Health Institute, Warsaw, Poland; 22 The University of Tokyo, Tokyo, Japan; 23 Department of Medicine, Teikyo University School of Medicine, Tokyo, Japan; 24 University of Miami Miller School of Medicine, Miami, Florida, USA; 25 Stanford University School of Medicine, Stanford, California, USA; 26 The Children’s Memorial Health Institute, Warsaw, Poland; 27 Department of Surgery and Science, Graduate School of Medical Sciences, Kyushu University, Fukuoka, Japan; 28 University Medical Center Hamburg-Eppendorf (UKE), Hamburg, Germany; 29 Liver Transplant Hepatology Unit, Division of Gastroenterology and Hepatology, Foundation IRCCS Ca’ Granda Ospedale Maggiore Policlinico, Milan, Italy; 30 University Medical Center Hamburg-Eppendorf, Hamburg, Germany; 31 Gastroenterology Service—IBIMA, University Hospital and CIBERehd, University of Málaga, Málaga, Spain; 32 Leiden University Medical Center, Leiden, The Netherlands; 33 Department of Medicine, University Hospital Erlangen, Friedrich-Alexander University Erlangen-Nürnberg, Erlangen, Germany; 34 Department of Gastroenterology and Hepatology, University Hospital Zürich, University of Zürich, Zürich, Switzerland; 35 Department of Gastroenterology and Hepatology, Graduate School of Medicine, Kyoto University, Kyoto, Japan; 36 University of Utah, Salt Lake City, Utah, USA; 37 University of Texas Southwestern Medical Center, Dallas, Texas, USA; 38 Department of Surgery, Oncology and Gastroenterology, University of Padova, Padova, Italy; 39 University of Padova, Padova, Italy; 40 Epatocentro Ticino, Università della Svizzera Italiana, Lugano, Switzerland; 41 UPMC Pediatric Liver Center, Palermo, Italy; 42 Hepatogastroenterology, Nutrition and Liver Transplant Unit, IRCCS Bambino Gesù Pediatric Hospital, Rome, Italy; 43 Henry Ford Health System, Detroit, Michigan, USA; 44 Pediatric Liver Unit, French National Reference Center for Rare Diseases Biliary Atresia and Genetic Cholestasis, Hôpital Necker, Université de Paris, Paris, France; 45 Gastroenterology–Hepatology–Nutrition Unit, Hôpital Necker–Enfants Malades, Paris, France; 46 Department of Gastroenterology and Hepatology, Amsterdam UMC, Vrije Universiteit Amsterdam, Amsterdam, The Netherlands; 47 Department of Biomedical Sciences, Humanitas University, Pieve Emanuele, Milan, Italy; 48 Division of Internal Medicine and Hepatology, Department of Gastroenterology, IRCCS, Humanitas Research Hospital, Rozzano, Milan, Italy; 49 Division of Gastroenterology, Department of Medicine, University of Alberta, Edmonton, Alberta, Canada; 50 King’s College Hospital NHS Foundation Trust, London, UK; 51 Center for Liver and Gastrointestinal Research and National Institute of Health Research (NIHR) Birmingham Biomedical Research Centre, University of Birmingham; Centre for Rare Disease and ERN Rare Liver Centre, Liver Transplant and Hepatobiliary Unit, University Hospital Birmingham NHS Foundation Trust, Birmingham, UK; 52 Division of Preventive Medicine, Department of Medicine, University of Alberta, Edmonton, Alberta, Canada

**Keywords:** artificial intelligence, autoimmune hepatitis, liver transplantation, machine learning, surveillance

## Abstract

**Background:**

Autoimmune hepatitis (AIH) is an important indication for liver transplantation (LT), but recurrence affects over 30% of recipients, threatening long-term survival. Current strategies to prevent recurrence and progressive graft fibrosis remain suboptimal, with limited evidence to guide selection of immunosuppressive regimens. We aimed to develop a dynamic, individualized, artificial intelligence–powered model for post-transplant recurrent AIH.

**Methods:**

We conducted a multicenter, retrospective cohort study of 706 patients who underwent LT for AIH between January 1987 and June 2020 at 33 centers in North America, South America, Europe, and Asia. We trained 4 predictive machine learning models—Logistic Regression, Random Forest, XGBoost, and Gradient Boost—to predict recurrent AIH (rAIH) using 62 clinical and laboratory variables, including demographic, biochemical features, and immunosuppressive drugs up to 1-year post-transplant. Feature importance was assessed using SHapley Additive exPlanations (SHAP) to enable interpretability at both individual and population levels.

Results:

AIH recurred in 16.5% of patients after LT. SHAP analysis identified younger age at LT, higher necroinflammatory activity in the explanted liver, and elevated MELD score at LT as key predictors of rAIH in the overall population. Tacrolimus-based therapy was associated with a lower risk of recurrence, while cyclosporine use conferred a higher risk. The addition of long-term prednisone to a regimen of tacrolimus and mycophenolate mofetil did not provide additional protective effect against rAIH.

**Conclusions:**

Our AI-powered clinical decision model provides personalized prediction of post-transplant rAIH. While it offers insight into modifiable and non-modifiable predictors, prospective validation is required before informing immunosuppressive decisions.

## INTRODUCTION

Liver transplantation (LT) is lifesaving for patients with end-stage liver disease due to autoimmune hepatitis (AIH).^1^ However, recurrent AIH (*r*AIH) has been reported to affect between 17% and 42% of patients who have undergone LT, and such variability can be explained by substantial variation in post-LT monitoring practices (such as protocol versus clinical indication biopsies), sample size, and duration of follow-up.^2^^–^^4^

Key risk factors for disease recurrence have been explored in large multicenter cohort studies, and include donor–recipient sex mismatch, high pre-transplant immunoglobulin G (IgG) levels, younger age at LT, and specific immunosuppressive regimens;^5^ however, the optimal long-term regimen for preventing recurrence remains unclear.^6^^,^^7^

Besides, in the setting of AIH, allograft fibrosis can advance insidiously, frequently needing invasive procedures for confirmation (liver biopsy), and in a subset of patients, may eventually lead to progressive fibrosis, cirrhosis, and end-stage liver disease requiring re-transplantation.^8^

Long-term low-dose corticosteroid (prednisolone 5–10 mg daily) use following LT has been suggested to lower the risk of *r*AIH without increasing the risk of septic complications or metabolic bone disease.^9^ However, this data is still controversial as the most recent guidance from the American Association for the Study of the Liver (AASLD) suggests that glucocorticoids can be stopped after LT, with subsequent monitoring for AIH recurrence.^10^

Moreover, the use of mycophenolate mofetil (MMF) within the first year post-LT was associated with approximately threefold higher risk of *r*AIH, as well as a higher incidence of post-LT septic complications.^4^ In addition, this study found no significant correlation between initial and 1-year post-LT MMF dosage in patients with and without *r*AIH, suggesting the use of alternative agents in addition to calcineurin inhibitors.^4^ Interestingly, post-LT treatment with azathioprine was associated with a lower risk of both *r*AIH and septic complications.^4^

However, since azathioprine was predominantly used in the 1980s and 1990s, the observed reduction in recurrence may be influenced by confounding factors from that era, such as shorter cold ischemia times, less severely ill patients, and shorter LT waiting periods.^4^

*r*AIH is linked to an increased risk of allograft fibrosis, graft loss, and decreased patient survival, thus highlighting the need for precise risk classification and customized post-transplant care techniques.^11^ In addition to conventional clinical and histological evaluation, developments in machine learning-based prediction techniques are showing promise for early identification and more accurate prognostication of graft outcomes and recurrent illness.^12^

Given this conflicting literature around the optimal immunosuppressive approach for patients transplanted for AIH, the aim of our study was to develop an individualized, dynamic prediction model for post-transplant *r*AIH using machine learning methods on longitudinal data. We, in addition, aimed to identify individualized immunosuppressive strategies to prevent AIH recurrence.

## METHODS

### Study population

This was a multicenter study with an original cohort that included 855 patients who received an LT from 1987 until 2020 with AIH as an indication, drawn from 33 centers across Asia, Europe, and North and South America. Patients included had a diagnosis of AIH based on the criteria outlined by both theAASLD and the European Association for the Study of the Liver (EASL).^10^^,^^13^ In addition, all patients met the criteria for probable or definitive diagnosis of AIH according to the International AIH simplified scoring system.^14^ From this original cohort, 119 patients with features of overlap syndrome with other chronic cholestatic autoimmune diseases [primary biliary cholangitis (PBC) or primary sclerosing cholangitis (PSC)] were excluded. We further excluded 30 patients who experienced recurrence within the first 12 months of LT, as the primary outcome of interest is recurrence beyond 1-year post-transplant. The final study cohort included 706 patients. All procedures performed in the study were in accordance with the ethical standards of the institutional and/or national research committee and with the 1964 Helsinki Declaration and its later amendments or comparable ethical standards. The study was approved by the University of Alberta Research Ethics Board.

### Clinical, immunological, and laboratory assessments

Based on clinical relevance and a pre-defined missingness cutoff of <20%, we included 62 features in model training to ensure reliability and clinical applicability of our findings. This approach allowed us to focus on the most representative and meaningful variables, reducing the risk of bias from missing data while maintaining data integrity for robust and interpretable results.

Variables included in the analysis encompassed demographic and clinical characteristics such as sex, ethnicity, age at AIH diagnosis and at the time of LT, time from AIH diagnosis to LT, and presence of concomitant autoimmune diseases. Immunological markers included pre-LT antinuclear antibodies (ANA), anti-smooth muscle antibodies (ASMA), anti-liver kidney microsome type 1 antibodies (anti-LKM1), and IgG levels.

Liver function tests, including alanine aminotransferase (ALT), aspartate aminotransferase (AST), alkaline phosphatase (ALP), and total bilirubin, were analyzed at the time of LT and 3, 6, and 12 months after LT. For each marker, both actual values and values normalized by the upper limit of normal (ULN) were used, with ULNs defined according to the center-specific reference ranges. The ULN for ALT ranged from 31 to 56 U/L, AST from 30 to 52 U/L, IgG from 16 to 18 g/L, and bilirubin from 18 to 22 μmol/L across different LT centers. We then created delta variables to capture the longitudinal trajectory of laboratory variables over time. Delta variables were calculated as the difference between baseline and each follow-up measurement, providing a more nuanced understanding of how dynamic changes in these parameters influence post-transplant outcomes.

In addition, we considered donor-related variables, including donor age and sex, as well as donor–recipient sex mismatch. Histological factors such as explant necroinflammatory activity and fibrosis stage, along with the occurrence of post-LT acute and chronic rejection episodes, were also incorporated into the analysis.^15^^,^^16^

### Immunosuppression after liver transplantation

The use of tacrolimus, cyclosporine, sirolimus, and other immunosuppressive medications, including MMF, azathioprine, and prednisone or prednisolone, during the initial post-LT period was assessed. To evaluate the use and trough levels of tacrolimus, cyclosporine, and mTOR inhibitors at the time of transplant and 1-year post-transplant, we generated binary indicator variables representing whether trough levels exceeded clinically significant thresholds. The thresholds used were for tacrolimus, >10 ng/mL at transplant and >6 ng/mL at 1 year; for cyclosporine, >224 ng/mL at transplant and >161 ng/mL at 1 year; and for mTOR inhibitors, >4.5 ng/mL at transplant and >6 ng/mL at 1 year. This approach allowed us to systematically evaluate the impact of higher immunosuppression exposure on clinical outcomes in a standardized way. Furthermore, we investigated the effect of immunosuppressive regimen complexity, defined by the use of 1, 2, or 3 agents, on the risk of AIH recurrence by including this as an ordinal predictor variable.

Each participating center’s complete medical records, including transplant databases, clinical notes, and, if accessible, pharmacy dispensing records, were used to gather immunosuppressive data. This approach depends on the methodology of our previously published cohort research,^4^ in which the International AIH research Group’s biannual meetings and thorough guidelines for every site were used to standardize data collection. To ensure internal consistency, centers cross-checked immunosuppressive regimens against recorded trough levels and treatment plans. The use of trough levels and binary indications for clinically relevant thresholds made it feasible to systematically evaluate immunosuppressive exposure across centers, regardless of how retrospective data might fail to detect all dose adjustments.

### Diagnosis of recurrent autoimmune hepatitis

Diagnosis of *r*AIH was made histologically and defined by the presence of typical histological findings of AIH, such as the presence of lymphocyte and/or plasmacytic portal inflammation with interface hepatitis, with or without the presence of rosettes of regenerating hepatocytes, and emperipolesis. For *r*AIH diagnosis, the liver biopsy had to be negative for findings suggestive of T-cell-mediated rejection (TCMR), including endothelieitis, lymphocytic cholangitis, or perivenular hepatocyte necrosis.

### Statistical and machine learning models

#### Model training, evaluation, and interpretability

The dataset was partitioned into 70% for model training and 30% for internal validation. A suite of statistical and machine learning models- logistic regression, random forest, XGBoost, and gradient boosting^17^^–^^20^ was implemented to compare predictive performance across diverse modeling strategies. To ensure the robustness and reproducibility of results, we conducted extensive hyperparameter optimization using five-fold cross-validation within the training set with the Adam optimizer and grid search (Supplemental Table S1, http://links.lww.com/HC9/C415). Given the inherent class imbalance in the outcome variables, class weighting strategies were applied to mitigate bias toward the majority class and to enhance the model’s learning capability for minority-class instances.

#### Machine learning classification model

The application of machine learning to predict rAIH has the potential to offer substantial advantages over traditional statistical approaches, particularly in its ability to manage complex, high-dimensional clinical datasets and uncover latent patterns associated with disease recurrence. Unlike classical regression models, ML algorithms including random forests (RF), gradient boosting (GB), and extreme gradient boosting (XGBoost) can capture non-linear interactions among diverse predictors such as biochemical markers, histopathological findings, and treatment-related variables, without requiring strict assumptions about data distribution or variable independence. Ensemble methods like RF, GB, and XGBoost further improve model robustness by reducing overfitting and enhancing generalizability through techniques such as bagging and boosting, while also offering interpretable outputs via feature importance metrics. By leveraging these capabilities, ML models enable more accurate and personalized recurrence risk stratification, which is essential for guiding immunosuppressive therapy and improving long-term patient outcomes. In doing so, machine learning not only elevates predictive performance but also offers clinically actionable insights that may remain obscured using conventional statistical tools, thereby advancing the principles of precision medicine in hepatology.^21^^,^^22^ The development and reporting of the prediction model adhered to the Transparent Reporting of a Multivariable Prediction Model for Individual Prognosis or Diagnosis–Artificial Intelligence (TRIPOD+AI) reporting guideline (Supplemental Information 1, http://links.lww.com/HC9/C415).

### Statistical analysis

Descriptive statistics were used to summarize the characteristics of the study population. Differences between the AIH recurrent and non-recurrent groups were assessed using two-sample *t* tests to evaluate statistical significance. In addition, chi-squared tests were used to compare group-wise differences in categorical variables.

After model training, we evaluated the generalizability and stability of each individual and ensemble classifier using 1000 bootstrap iterations on the internal validation set. Performance was assessed based on key metrics, including Area Under the Receiver Operating Characteristic curve (AUROC), sensitivity, and specificity with corresponding 95% confidence intervals (95% CIs). Using 1000 bootstrap iterations, we performed calibration analysis using the Nomogram and the Brier score. Predicted probabilities of AIH recurrences were grouped into 12 equal-width bins, and the mean predicted probability was compared against the observed event rate (actual AIH recurrence proportion) within each bin. Across all 1000 iterations, bin-level statistics were aggregated to yield a bootstrap-averaged calibration curve with 95% confidence intervals (2.5th–97.5th percentiles). A LOWESS (locally weighted scatterplot smoothing) curve with a bandwidth fraction of 0.4 was overlaid to illustrate the overall trend. Calibration accuracy was further quantified using the Brier score, reported as mean and standard deviation (SD) across bootstrap iterations.

To enhance model interpretability and support clinical translation, we conducted SHapley Additive exPlanations (SHAP) analysis at both the population and individual levels.^23^ At the population level, Shapley values enabled us to identify the most influential predictors driving overall model performance. This helped generate clinically meaningful insights into risk stratification and potential intervention targets across the cohort.

At the individual level, SHAP analysis provided personalized feature attributions for each randomly sampled patient, allowing us to visualize how specific variables influenced the predicted risk in a case-specific context. This individualized interpretability supports transparent and explainable AI, empowering clinicians to better understand and trust the model’s recommendations in real-world scenarios.

By integrating both macro- and micro-level interpretability, this dual approach offers a comprehensive picture—bridging global insights with patient-specific rationale—and enhances the model’s utility for precision medicine applications.

### Code availability

All analyses were conducted using Python 3.10.14. Data manipulation and numerical operations were performed using NumPy (v2.0.1) and Pandas (v2.2.2). Model development, preprocessing, feature selection, and evaluation were implemented using scikit-learn (v1.5.1), with additional gradient boosting models built using XGBoost (v2.1.1).

## RESULTS

### Study population characteristics

**FIGURE 1:**
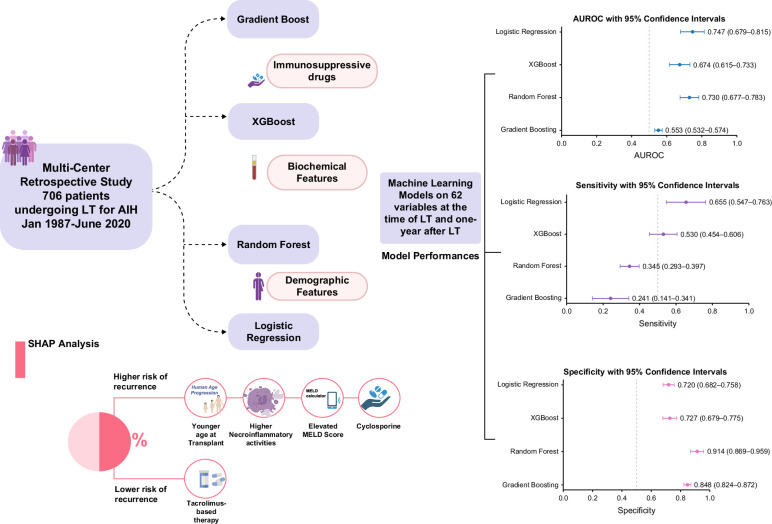
Study design, machine learning workflow, model performance, and SHAP-based feature interpretation for prediction of autoimmune hepatitis recurrence after liver transplantation. Abbreviations: AIH, autoimmune hepatitis; AUROC, area under the receiver operating characteristic curve; LT, liver transplantation; SHAP, SHapley Additive exPlanations; XGBoost, extreme gradient boosting.

The study included 706 patients who received an LT for end-stage liver disease secondary to AIH, excluding those who developed rAIH within the first year after LT. Females comprised 76.6% of the cohort. The mean age at AIH diagnosis was 34.4 (95% CI 33.1–35.7) years, and the mean age at the time of LT was 41.9 (95% CI 40.7–43.2) years. Of the total cohort, 589 patients (83.4%) did not experience AIH recurrence, and 117 (16.6%) developed rAIH more than 1 year after LT. Ethnicity varied substantially, with Caucasians representing over 63% of the cohort. Patients with AIH recurrence were significantly younger at both diagnosis (25.7 vs. 35.9 years, *p*<0.001) and LT (33.8 vs. 43.6 years, *p*<0.001) compared with those who did not experience recurrence. Blood type O was more prevalent among those with recurrence (57.3% vs. 44.1%, *p*=0.010) (Figure 1).

Certain pre-LT features of liver decompensation were also more common in the recurrence group, including ascites (63.0% vs. 53.8%, *p*=0.011) and variceal hemorrhage (18.3% vs. 26.5%, *p*=0.002). Donor–recipient sex mismatch was significantly higher in patients with recurrence (63.0% vs. 46.2%, *p*=0.004).

The distributions of recurrence and rejection according to 1-year tacrolimus trough levels are shown in Supplemental Figures S1A, B, http://links.lww.com/HC9/C415. Among 245 patients with a 1-year tacrolimus trough level above 6 ng/mL, 21.2% experienced rAIH, while 78.8% did not. Similarly, among 241 recipients with a 1-year tacrolimus trough level in the same range, rejection occurred in 36.9% of cases.

Pre-transplant immunoglobulin levels showed notable variation, with lower IgA levels (0.8×ULN vs. 1.1, *p*<0.001) and higher IgG levels (1.7×ULN vs. 1.4, *p*=0.008) in the recurrence group. Immunosuppressive regimens initially after transplant also differed significantly: cyclosporine use was more common among recurrence cases (24.3% vs. 8.3%, *p*<0.001), while tacrolimus use was less frequent (59.0% vs. 81.8%, *p*<0.001).

These findings underscore key demographic, clinical, and therapeutic factors describing cohorts with and without AIH recurrence following liver transplantation (Table 1).

**TABLE 1:** Demographic, clinical, and treatment-related variables for the overall cohort, depending on AIH recurrence status after liver transplantation

Variables	Category	Missing (N)	AIH LT recipients (N=706)	No recurrence (N=589)	Recurrence (N=117)	*p*
Sex, n (%)	Female	0	541 (76.6)	444 (75.4)	97 (82.9)	0.102
Ethnicity, n (%)	Caucasian	44	451 (63.9)	394 (66.9)	57 (48.7)	**<0.001** ^a^
	Asian		56 (7.9)	53 (9.0)	3 (2.6)	
	Black		22 (3.1)	18 (3.1)	4 (3.4)	
	Other		133 (18.8)	96 (16.3)	37 (31.6)	
	Missing		44 (6.2)	28 (4.8)	16 (13.7)	
Age at diagnosis, mean (95% CI)		124	34.3 (32.8–35.7)	35.9 (34.3–37.5)	25.7 (22.6–28.9)	**<0.001** ^a^
Age at transplant, mean (95% CI)		0	41.9 (40.7–43.2)	43.6 (42.2–44.9)	33.8 (30.9–36.7)	**<0.001** ^a^
AIH type, n (%)	1.0	136	537 (76.1)	446 (75.7)	91 (77.8)	0.540
	2.0		33 (4.7)	26 (4.4)	7 (6.0)	
	Missing		136 (19.3)	117 (19.9)	19 (16.2)	
Blood type, n (%)	A	0	250 (35.4)	216 (36.7)	34 (29.1)	**0.010** ^b^
	B		92 (13.0)	84 (14.3)	8 (6.8)	
	AB		37 (5.2)	29 (4.9)	8 (6.8)	
	O		327 (46.3)	260 (44.1)	67 (57.3)	
Ascites , n (%)	Yes	69	434 (61.5)	371 (63.0)	63 (53.8)	**0.011** ^c^
	Missing		69 (9.8)	49 (8.3)	20 (17.1)	
Concomitant autoimmune disease, n (%)	Yes	20	143 (20.3)	113 (19.2)	30 (25.6)	0.125
	Missing		20 (2.8)	19 (3.2)	1 (0.9)	
Variceal hemorrhage, n (%)	Yes	76	139 (19.7)	108 (18.3)	31 (26.5)	**0.002** ^b^
	Missing		76 (10.8)	56 (9.5)	20 (17.1)	
Hepatic encephalopathy, n (%)	Yes	71	338 (47.9)	289 (49.1)	49 (41.9)	**0.018** ^c^
	Missing		71 (10.1)	51 (8.7)	20 (17.1)	
Prednisone, n (%)	Yes	0	479 (67.8)	398 (67.6)	81 (69.2)	0.808
Azathioprine, n (%)	Yes	0	324 (45.9)	267 (45.3)	57 (48.7)	0.569
Budesonide, n (%)	Yes	0	18 (2.6)	14 (2.4)	4 (3.5)	0.514
Mycophenolic acid, n (%)	Yes	0	57 (8.1)	49 (8.3)	8 (6.8)	0.725
Transplant type, n (%)	1.0	77	45 (6.4)	35 (5.9)	10 (8.5)	**0.003** ^b^
	2.0		443 (62.7)	357 (60.6)	86 (73.5)	
	3.0		141 (20.0)	132 (22.4)	9 (7.7)	
	Missing		77 (10.9)	65 (11.0)	12 (10.3)	
Anastomosis type, n (%)	1.0	112	55 (7.8)	40 (6.8)	15 (12.8)	0.174
	2.0		532 (75.4)	449 (76.2)	83 (70.9)	
	3.0		7 (1.0)	6 (1.0)	1 (0.9)	
	Missing		112 (15.9)	94 (16.0)	18 (15.4)	
Explant biopsy fibrosis stage, n (%)	0.0	123	0 (0.0)	0 (0.0)	0 (0.0)	**<0.001** ^a^
	1.0		34 (4.8)	31 (5.3)	3 (2.6)	
	2.0		26 (3.7)	25 (4.2)	1 (0.9)	
	3.0		46 (6.5)	38 (6.5)	8 (6.8)	
	4.0		477 (67.6)	408 (69.3)	69 (59.0)	
	Missing		123 (17.4)	87 (14.8)	36 (30.8)	
Presence of plasma cells on the explant biopsy, n (%)	Yes	123	281 (39.8)	239 (40.6)	42 (35.9)	**<0.001** ^a^
	Missing		123 (17.4)	87 (14.8)	36 (30.8)	
Explant biopsy necroinflammatory activity, n (%)	0.0	123	45 (6.4)	38 (6.5)	7 (6.0)	**<0.001** ^a^
	1.0		164 (23.2)	150 (25.5)	14 (12.0)	
	2.0		166 (23.5)	141 (23.9)	25 (21.4)	
	3.0		112 (15.9)	92 (15.6)	20 (17.1)	
	4.0		96 (13.6)	81 (13.8)	15 (12.8)	
	Missing		123 (17.4)	87 (14.8)	36 (30.8)	
Donor sex, n (%)	Female	127	300 (42.5)	248 (42.1)	52 (44.4)	0.363
	Missing		127 (18.0)	102 (17.3)	25 (21.4)	
Donor age, mean (95% CI)		119	41.7 (40.3–43.1)	42.3 (40.8–43.8)	38.7 (34.9–42.4)	0.080
Recipient–donor sex mismatch, n (%)	Yes	127	283 (40.1)	225 (38.2)	58 (49.6)	**0.008** ^b^
	Missing		127 (18.0)	102 (17.3)	25 (21.4)	
IgA (g/L), mean (95% CI)		292	1.1 (0.98–1.12)	1.1 (1.00–1.17)	0.8 (0.72–0.95)	**<0.001** ^a^
IgG (g/L), mean (95% CI)		357	1.4 (1.36–1.50)	1.4 (1.31–1.44)	1.7 (1.48–2.00)	**0.008** ^b^
IgM (g/L), mean (95% CI)		348	1.0 (0.93–1.12)	1.0 (0.92–1.14)	1.0 (0.77–1.20)	**<0.001** ^a^
ALT (U/L), mean (95% CI)		103	201.1 (164.8–237.5)	205.1 (164.5–246.8)	178.1 (124.0–232.2)	0.435
ALP (U/L), mean (95% CI)		110	203.7 (189.9–217.5)	201.9 (189.5–214.3)	214.2 (152.9–275.5)	0.696
AST, (U/L), mean (95% CI)		86	222.0 (176.1–268.0)	227.0 (173.9–280.0)	194.0 (132.8–255.2)	0.422
Bilirubin (mg/dL or µmol/L), mean (95% CI)		77	189.7 (164.1–215.4)	183.4 (160.2–206.5)	226.3 (113.9–338.7)	0.460
Creatinine (mg/dL or µmol/L), mean [95% CI]		84	104.1 (76.9–131.2)	90.2 (84.1–96.3)	183.8 (1.61–365.9)	0.311
INR, mean (95% CI)		89	2.1 (2.01–2.20)	2.1 (1.96– 2.14)	2.4 (2.04–2.79)	0.063
MELD score, mean (95% CI)		104	22.5 (21.71–23.25)	22.4 (21.55–23.10)	23.1 (20.91–25.28)	0.539
Tacrolimus, n (%)	Yes	0	551 (78.0)	482 (81.8)	69 (59.0)	**<0.001** ^a^
Prednisone, n (%)	Yes	0	536 (75.9)	456 (77.4)	80 (68.4)	0.111
	Missing	4	4 (0.6)	3 (0.5)	1 (0.9)	
AZA/MMF, n (%)	Yes	0	457 (64.7)	393 (66.7)	64 (54.7)	0.054
Cyclosporine, n (%)	Yes	0	76 (11.0)	48 (8.3)	28 (24.3)	**<0.001** ^a^
mTOR inhibitor, n (%)	Yes	0	14 (2.0)	14 (2.4)	0.0 (0.0)	0.143
Immunosuppression trough level 1 year after transplant						
Tacrolimus (ng/mL)		346	7.6 (7.23–7.89)	7.3 (6.98– 7.65)	8.8 (7.76–9.86)	**0.008** ^b^
Cyclosporine (ng/mL)		664	239.1 (168.26–309.99)	265.6 (153.69–377.48)	196.1 (142.20–250.05)	0.254
mTOR inhibitor (ng/mL)		683	5.9 (4.54–7.16)	5.7 (4.37–7.07)	8.7 (N/A)	0.349

*Note:* Categorical variables coded numerically are defined as follows: Transplant type: 1=living donor liver transplantation, 2=deceased donor liver transplantation, 3=combined or split graft transplantation. Anastomosis type: 1=duct-to-duct biliary anastomosis, 2=Roux-en-Y hepaticojejunostomy, 3=other techniques. Explant biopsy fibrosis stage was graded according to standard histological staging systems, where 0=no fibrosis, 1–2=mild to moderate fibrosis, 3=advanced fibrosis, and 4=cirrhosis. Necroinflammatory activity was graded on a scale of 0–4, with higher scores indicating greater inflammatory activity.

^a^ *p*≤0.001.

^b^ *p*≤0.01.

^c^ *p*≤0.05.

Abbreviations: AIH, autoimmune hepatitis; ALP, alkaline phosphatase; ALT, alanine transaminase; AST, aspartate aminotransferase; AZA, azathioprine; CI, confidence interval; Ig: immunoglobulin; INR, international normalized ratio; LT, liver transplantation; MELD, Model for End-Stage Liver Disease; MMF, mycophenolate mofetil; mTOR, mammalian target of rapamycin; N/A, not applicable.

### Model performances

#### Logistic regression

Among the trained models, logistic regression achieved the highest overall discriminative performance among other models, with an AUROC of 0.747 (95% CI 0.679–0.815), sensitivity of 0.655 (95% CI 0.547–0.763), and specificity of 0.720 (95% CI 0.682–0.758) (Table 2). This balance between sensitivity and specificity suggests that they are particularly well-suited for applications where both identifying true positives and minimizing false positives [41 false positives (FPs) and 5 false negatives (FNs) out of 212 test patients] is crucial, with logistic regression showing the strongest overall predictive performance.

**TABLE 2:** Key performance metrics of machine learning algorithms used in the study

Model	AUROC (95% CI)	Sensitivity (95% CI)	Specificity (95% CI)
Logistic Regression	0.747 (0.679–0.815)	0.655 (0.547–0.763)	0.720 (0.682–0.758)
XGBoost	0.674 (0.615–0.733)	0.5^1^30 (0.454–0.606)	0.727 (0.679–0.775)
Random Forest	0.730 (0.677–0.783)	0.914 (0.869–0.959)	0.345 (0.293–0.397)
Gradient Boosting	0.553 (0.532–0.574)	0.241 (0.141–0.341)	0.848 (0.824–0.872)

Abbreviations: AUROC, area under the receiver operating characteristic curve; CI, confidence interval; XGBoost, Extreme Gradient Boosting.

#### XGBoost

XGBoost also offered a balanced profile, with an AUROC of 0.674 (95% CI 0.615–0.733), sensitivity of 0.530 (95% CI 0.454–0.606), and specificity of 0.727 (95% CI 0.679–0.775), suggesting a moderate ability to identify both true positive and true negative cases.

#### Random forest

The random forest model yielded a moderate AUROC of 0.730 (95% CI 0.677–0.783) and the highest specificity among all models at 0.914 (95% CI 0.869–0.959), but its sensitivity was relatively low at 0.345 (95% CI 0.293–0.397), indicating a greater tendency to miss positive cases. This conservative behavior may be preferable in scenarios where avoiding false positives is critical; for example, when a recurrent disease is highly suspicious but needs confirmation.

#### Gradient boosting

In contrast, the gradient boosting model showed the weakest overall performance, with an AUROC of 0.553 (95% CI 0.532–0.574), low sensitivity of 0.241 (95% CI 0.141–0.341), and high specificity of 0.848 (95% CI 0.824–0.872). This indicates limited discriminative power and a strong bias toward predicting negative cases.

#### Model calibration, risk stratification, and threshold optimization

As the LR model demonstrated superior discriminative performance, we evaluated the clinical value of logistic regression by examining the predicted probability distributions throughout the test set (n=212).

Instead of clustering around uncertain probabilities close to 0.5, Supplemental Figure S2, http://links.lww.com/HC9/C415, showed the distribution of predicted probabilities, which is a well-calibrated model with meaningful risk stratification (mean: 0.437, median: 0.420, SD: 0.141). In all, 2.8% of patients had extremely low predicted probabilities (0.0–0.2), 40.6% had low-to-moderate risk (0.2–0.4), 43.9% (n=93) had moderate-to-high risk (0.4–0.6), and 12.7% (n=27) had high risk (0.6–0.8). None of the patients was higher than 0.8. This wide range of expected probability (0.00–0.08) shows that the model does more effectively differentiate patient risk than a binary classification.

The most effective thresholds varied depending on the model architecture: logistic regression performed best among other models at θ=0.505 (AUROC: 0.836), gradient boosting at θ=0.495 (AUROC: 0.905), random forest at θ=0.859 (AUROC: 0.832), and XGBoost at θ=0.505 (AUROC: 0.773). The optimal threshold of 0.505 for the logistic regression model maximized the F1 score while balancing sensitivity (0.655) and specificity (0.720), offering a clinically appropriate risk classification decision boundary (Supplemental Figure S3A, http://links.lww.com/HC9/C415).

The calibration plot of logistic regression demonstrates good overall agreement between the nomogram-predicted probabilities and the observed AIH recurrence rates across the probability spectrum. The bootstrap-averaged LOWESS curve closely tracks the diagonal line of perfect calibration, with the binned data points falling near or on the identity line across all risk strata from the low-risk through to the high-risk range. The model shows no marked systematic overestimation or underestimation of recurrence risk. The Brier score was 0.108 ± 0.010, indicating good probabilistic accuracy given the baseline recurrence rate in this cohort. The 95% confidence intervals widen at higher predicted probabilities, reflecting the smaller number of patients in the high-risk bins and correspondingly greater uncertainty in those strata, which is expected in a dataset of this size. Overall, these results support that the logistic regression model is well-calibrated across the full range of predicted risks (Supplemental Figure S3B, http://links.lww.com/HC9/C415).

### Features associated with AIH recurrence

To identify key drivers of the model’s predictions and align them with clinical knowledge, while providing actionable insights that validate the relevance and reliability of the findings, we conducted SHAP analysis of the best-performing model, logistic regression, on the test set.

**FIGURE 2:**
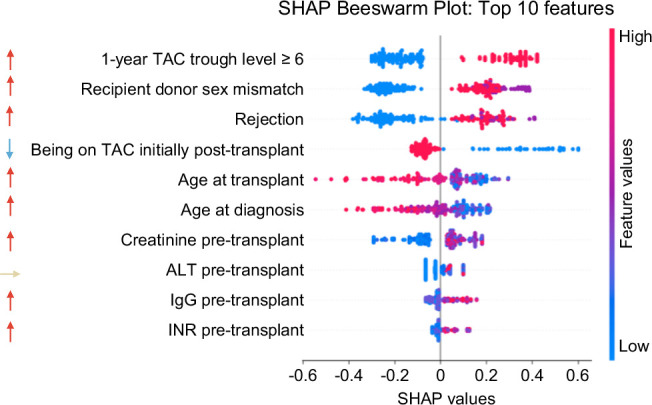
SHAP bee swarm plot for logistic regression model predicting AIH recurrence risk after liver transplantation. Positive SHAP values (right of the *y*-axis) indicate stronger contribution toward recurrence prediction, while negative values (left of the *y*-axis) indicate protective effects. Data point colors reflect feature values, as indicated by the color bar. For binary variables, red denotes “yes” and blue denotes “no”. For continuous variables, color intensity corresponds to magnitude, with a gradient scale representing low to high values. Abbreviations: AIH, autoimmune hepatitis; ALT, alanine aminotransferase; IgG, Immunoglobulin G; INR, International Normalized Ratio; SHAP, SHapley Additive exPlanations; TAC, tacrolimus.

The SHAP bee swarm plot (Figure 2) offers a visual summary of the top 10 features that most strongly influence the model’s predictions of AIH recurrence risk in LT recipients. It conveyed both the direction and magnitude of each feature’s contribution, with SHAP values quantifying their relative importance. Among the features, a 1-year post-LT tacrolimus level ≥ 6 ng/mL showed the strongest positive association with recurrence risk, as indicated by the concentration of high SHAP values on the positive side of the axis. Interestingly, the immediate post-LT use of tarcrolimus demonstrates a bidirectional effect: in some cases, it was associated with reduced recurrence risk (negative SHAP values), while in others, it corresponded to an increased risk. This variability likely reflects marked individual differences in immunosuppressive response and tolerance.

Recipient–donor sex mismatch and history of rejection also displayed moderately positive SHAP values, implying a higher likelihood of recurrence. Demographic variables such as age at LT and age at diagnosis exhibited heterogeneous effects. Younger recipients had a higher recurrence risk, whereas older recipients appeared to have a lower risk. Among pre-LT biochemical markers, elevated creatinine and International Normalized Ratio (INR) levels were linked to the increased recurrence risk, likely indicating a negative impact of the compromised hepatic and renal function at the time of transplant. In addition, higher IgG levels were associated with an increased risk of *r*AIH.

To further illustrate the model’s capability in delivering personalized, patient-specific predictions and interpretations, we generated SHAP value analyses for individual patients to highlight the model’s capacity to delineate patient-specific contributions of predictive features (Supplemental Figure S4, http://links.lww.com/HC9/C415). The plots display the top five features with the highest absolute SHAP values, representing the most influential contributors to the predicted probability scores.

The individual SHAP evaluations for four patients showed distinct patterns in the ways that characteristics affected the model’s ability to predict the recurrence of AIH. In this case, a higher anticipated probability means a higher chance of recurrence, whereas a lower probability means a lower chance.

For Patient 63, the predicted class is *no recurrence*, with a probability of 0.4330. Protective factors contributing to this lower risk include a 1-year tacrolimus trough level below 6 and older age at transplant (57 years). Among the top 5 features, having experienced a rejection episode, not being on tacrolimus immediately post-LT, and not belonging to a non-Caucasian ethnicity increase the predicted risk. However, the overall classification remains *no recurrence*, due to the dominant influence of the protective features.

Similarly, Patient 116 is also classified as *no recurrence*, with a probability of 0.3874. The patient has a 1-year tarcolimus trough level above 6 ng/mL and an elevated INR of 2.91 pre-transplant, both of which contribute to a higher risk. However, these risk factors are balanced and compensated for by several protective features. The patient did not experience rejection episodes and had no sex mismatch with the donor, both of which significantly reduce the predicted risk. In addition, the patient’s blood type is not O, which appears to have a protective effect on this model. The combination of these protective factors ultimately outweighs the risk-enhancing features, resulting in a *no recurrence* classification.

In contrast, Patient 61 is predicted to have a *recurrence* outcome, with a probability of 0.5333. The primary contributor to this classification is that the patient did not receive tacrolimus initially after LT. Despite some mitigating features, such as older age at LT, the dominant effect of risk-enhancing variables tips the overall prediction toward *having recurrence*, illustrating the complex interplay of contributing factors.

Patient 8 shows a more confident recurrence prediction, with a probability of 0.7414. Key features increasing recurrence risk in this case include younger age at both diagnosis and transplant, sex mismatch with the donor, history of rejection, and a high tacrolimus trough level at 1 year after LT. These factors strongly drive the prediction toward a higher risk classification. Notably, this patient has no protective features working in their favor, as all measured variables contribute to increased risk.

Overall, these SHAP analyses underscore the individualized nature of model predictions, revealing how different combinations of clinical features can uniquely influence outcomes. Recurrent patterns across cases include the association of rejection episodes and elevated tacrolimus trough levels with increased recurrence risk, while initial use of tacrolimus appears protective.

## DISCUSSION

In this multicenter, international study, we developed and validated a dynamic, interpretable machine learning model to predict individualized risk of recurrent AIH post-transplant. Our study considered longitudinal data from 706 LT recipients transplanted for AIH across diverse settings and underscores the importance of personalized, data-driven approaches to risk stratification and therapeutic decision-making in the care of LT recipients.

Several important predictors of recurrent AIH were found by our machine learning model. Recurrence risk was most strongly associated with a 1-year post-transplant tacrolimus trough level ≥6 ng/mL, which was followed by recipient–donor sex mismatch and rejection history. In line with the clinical perception that younger patients, who frequently have more aggressive AIH disease, are at higher risk of recurrence, a younger age at transplant also emerged as a significant predictor.^24^ Elevated levels of creatinine, INR, ALT, and IgG were among the pre-transplant biochemical indicators linked to an increased risk of recurrence. Higher hepatocyte necrosis and active necroinflammatory activity are directly indicated by elevated ALT,^25^ indicating that more severe necroinflammation on explant is a significant predictor of recurrence. Hepatic dysfunction and the severity of the disease at transplant are indicated by elevated creatinine and INR, which are components of the MELD score.^25^ An elevated MELD score at transplant is also a marker of disease severity at transplant, reflecting steroid non-responsiveness/refractory disease before transplant.^26^ Moreover, increased autoimmune activity is reflected in elevated IgG levels.

Most importantly, our study highlights the varying impact of immunosuppressive strategies on the risk of rAIH. The optimal immunosuppression regimen to protect against rAIH has been a matter of debate, and one cannot use a one-size-fits-all approach. We discovered that tacrolimus-based immunosuppression was protective overall, especially immediately post-transplant. Higher tacrolimus trough levels at 1 year after transplant, on the other hand, were correlated with recurrence; this is probably not an immediate consequence of tacrolimus exposure but rather an increase in immunosuppression in patients with increased immunologic activity. One-year tacrolimus levels should be considered as a clinical risk marker identifying patients at increased immunologic risk rather than evidence that higher troughs lead to recurrent disease because early recurrence (<1 year) was excluded, and longitudinal liver biochemistry was computed independently. This contrasted with cyclosporine, which was associated with substantially higher risk of rAIH and consistent with prior literature showing that tacrolimus is an effective second-line treatment for AIH,^27^ and higher rates of TCMR episodes after LT.^28^^,^^29^ This finding is consistent with previous meta-analyses by Chen et al^30^ that highlight the value of customized immunosuppressive techniques in improving long-term results for AIH patients following transplantation.

The role of corticosteroids in preventing rAIH has been the subject of debate. Our study findings suggest that the presence of prednisone in combination regimens (eg, tacrolimus + MMF + prednisone) did not confer added benefit over tacrolimus-based dual therapy, aligning with emerging literature that routine long-term prednisone may not be necessary for all AIH recipients. According to the AASLD, there is no evidence to support the claim that ongoing corticosteroid usage and steroid removal following transplantation for AIH significantly differ in terms of rAIH rates, graft loss, or patient survival. Although the results do not support routine long-term prednisone for all recipients, the quality of the evidence is low.^10^

While the AASLD 2019 guidelines initially suggested limited evidence regarding steroid withdrawal post-transplant, this recommendation was based on a systematic review that included only 4 publications with 2 observational studies meeting criteria for quantitative meta-analysis. A subsequent dedicated systematic review and meta-analysis by Vierling et al^31^ provided more robust evidence from ~1050 patients across multiple studies. This larger evidence base demonstrated that steroid withdrawal post-transplant for AIH does not significantly increase rates of recurrent AIH, graft loss, or mortality compared with steroid continuation, supporting the safety of steroid withdrawal in appropriately selected patients.

Our results may be explained by the strong suppression of alloimmune and autoimmune activity by current calcineurin inhibitor-based regimens, especially tacrolimus, and the absence of further immunologic benefit from long-term low-dose corticosteroids in this instance. In addition, the negative consequences of prolonged corticosteroid use, such as metabolic, viral, and bone issues, highlight how crucial it is to reduce steroid exposure whenever feasible.^10^^,^^32^ Therefore, this growing knowledge of post-transplant immunosuppression in AIH emphasizes the value of customized strategies as opposed to standard operating procedures. Particularly for higher-risk patients who might benefit from closer monitoring during steroid withdrawal or maintenance of steroid therapy, our machine learning model’s capacity to identify patient-specific risk factors can assist in informing these tailored decisions regarding steroid continuation or withdrawal.

The use of SHAP analysis at the population and individual levels provides interpretability and potential for tailored preventive and immunosuppressive strategies based on patient-specific drivers of rAIH. We illustrate this tailored approach through the patient examples in the results section, revealing the convergence of clinical factors that together modulate risk. Therefore, the model output can inform closer monitoring or adjustments to therapy even in the absence of overt biochemical derangements. Post-transplant monitoring could be guided by high predicted risk profiles, which may include factors such as elevated IgG levels, a younger age at the time of transplantation, or previous episodes of rejection. Our model’s probability predictions, which range from 0.0 to 0.8, facilitate clinically actionable risk stratification. These higher-risk patients would potentially benefit from intensified surveillance or preventive modifications to their immunosuppressive regimens. Notably, these results are in line with earlier multicenter research and meta-analyses that found that advanced liver dysfunction, significant pre-transplant disease activity, and younger age are risk factors for recurrence and unfavorable outcomes.^4^^,^^30^ The alignment of our personalized predictions with known risk factors described in the literature reinforces the clinical relevance of the proposed model outputs.

Although our results demonstrated the promising implications, they should be interpreted carefully due to the moderate model performance. Out of 212 patients in the independent test set, 41 patients were classified as high risk without developing recurrence (false positive); 5 patients who recurred later were not identified (false negative). This trade-off in the context of immunosuppression management is clinically important. As unnecessary escalation of immunosuppression may lead to increased risks of infection, metabolic complications, and malignancy, meanwhile, missed high-risk patients may lead to preventable graft injury.

As a result, the model should be interpreted as a complementary risk-stratification tool to monitor intensity and shared decision-making rather than as a prescriptive trigger for automatic immunosuppression modification.

### Limitations

The retrospective nature of our study limits our ability to establish causality. While prednisone was identified as a risk factor for rAIH, though it is known to be protective for inflammatory activity, this likely reflects confounding by indication, as patients’ higher disease activity will remain on prednisone. The use of MMF and azathioprine varies, which could reflect physician bias rather than the intrinsic medication benefit itself. Although we collected data on immunosuppressive regimens using standardized forms and validated them against trough levels, some dose adjustments may not be captured through retrospective documentation, which could introduce residual misclassification bias. As another limitation of this paper, there was inadequate follow-up and inconsistent histological or imaging assessment; outcomes such as graft fibrosis or re-transplantation could not be investigated. To properly capture these late post-transplant complications, longer-term investigations are required. Finally, there may be interinstitutional variability in histopathologic grading due to a lack of standardization.

## CONCLUSION

In summary, our multicenter, international study generates a robust, personalized ML model for personalized prediction of recurrent AIH. By providing independent weights of each modifiable and non-modifiable feature in risk prediction, we offer a foundation for individualized immunosuppressive strategies in AIH LT recipients. Integration of this AI model into the clinical workflow could reduce the burden of recurrent AIH and optimize long-term graft survival. Future prospective evaluation of this model in real-world transplant care, with the addition of variables to fine-tune performance, will be important to inform personalized care for AIH LT recipients.

## Supplementary Material
